# Asymmetrical sexual isolation but no postmating isolation between the closely related species *Drosophila suboccidentalis and Drosophila occidentalis*

**DOI:** 10.1186/s12862-015-0328-y

**Published:** 2015-03-12

**Authors:** Nicholas J Arthur, Kelly A Dyer

**Affiliations:** Department of Genetics, University of Georgia, Athens, GA 30602 USA

**Keywords:** Speciation, Reproductive isolation, Hybridization, Prezygotic, Postzygotic, *Drosophila*

## Abstract

**Background:**

During the speciation process several types of isolating barriers can arise that limit gene flow between diverging populations. Studying recently isolated species can inform our understanding of how and when these barriers arise, and which barriers may be most important to limiting gene flow. Here we focus on *Drosophila suboccidentalis* and *D. occidentalis*, which are closely related mushroom-feeding species that inhabit western North America and are not known to overlap in geographic range. We investigate patterns of reproductive isolation between these species, including premating, postmating prezygotic, and postzygotic barriers to gene flow.

**Results:**

Using flies that originate from a single population of each species, we find that the strength of premating sexual isolation between these species is asymmetric: while *D. occidentalis* females mate with *D. suboccidentalis* males at a reduced but moderate rate, *D. suboccidentalis* females discriminate strongly against mating with *D. occidentalis* males. Female hybrids will mate at high rates with males of either species, indicating that this discrimination has a recessive genetic basis. Hybrid males are accepted by females of both species. We do not find evidence for postmating prezygotic or postzygotic isolating barriers, as females use the sperm of heterospecific males and both male and female hybrids are fully fertile.

**Conclusions:**

Premating isolation is substantial but incomplete, and appears to be the primary form of reproductive isolation between these species. If these species do hybridize, the lack of postzygotic barriers may allow for gene flow between them. Given that these species are recently diverged and are not known to be sympatric, the level of premating isolation is relatively strong given the lack of intrinsic postzygotic isolation. Further work is necessary to characterize the geographic and genetic variation in reproductive isolating barriers, as well as to determine the factors that drive reproductive isolation and the consequences that isolating barriers as well as geographic isolation have had on patterns of gene flow between these species.

**Electronic supplementary material:**

The online version of this article (doi:10.1186/s12862-015-0328-y) contains supplementary material, which is available to authorized users.

## Background

The process of speciation generates barriers to reproduction that prevent gene flow between diverging populations [1-3]. These isolating mechanisms are distinguished based on the stage of sexual reproduction at which they act. Prezygotic isolating barriers prevent the initial generation of a hybrid zygote. One of the most important prezygotic barriers to reproduction in sexually reproducing animal species is behavioral isolation, which includes factors that reduce the attraction between heterospecific individuals, thus preventing mating [1]. If mating does occur, postmating prezygotic barriers can limit the generation of hybrid offspring [1,4]. These barriers include the precedence of conspecific sperm over heterospecific sperm in female reproductive tracts (e.g. [5]), incompatibilities between sperm and egg surface proteins that prevent fertilization (e.g. [6]), and reduced female survival due to exposure to heterospecific sperm toxins (e.g. [7]). Postzygotic barriers to reproduction occur after hybrid offspring are produced, and typically involve genetic incompatibilities within the hybrid genome that result in a loss of viability or fertility of the hybrid offspring (e.g. [8-10]).

Multiple isolating mechanisms that promote reproductive isolation can exist between even incipient species (e.g. [11-19]). Thus, in order to understand how divergence is maintained between two species one must quantify both prezygotic and postzygotic barriers to reproduction. By dissecting these barriers it is possible to determine the relative strength of each, which can establish how gene flow between populations is prevented as well as indicate which barriers arose first. Emerging patterns across species may also shed light on the importance of natural selection during the speciation process. For instance, across species of *Drosophila* levels of both behavioral isolation and postzygotic isolation increase as the genetic divergence increases [20,21]. When taxa are allopatric, premating and postmating barriers tend to accumulate at similar rates. However, when taxa are sympatric, behavioral isolation generally increases faster than postzygotic isolation, which is thought to be due to natural selection reinforcing species boundaries to strengthen premating isolation [20,21].

In this study, we focus on two closely related species of *Drosophila*, *D. suboccidentalis* and *D. occidentalis*. These species are members of the quinaria group*,* which contains around 30 species and is about 10–15 million years old [22,23]. *D. suboccidentalis* is abundant and broadly distributed in northwestern North America, including Washington, Oregon, Colorado, North and South Dakota, Idaho, Utah, British Columbia, and Alberta. There are also collecting records of this species from the mountains of central Mexico [24]; its range may be contiguous south to this region, though it has not been collected recently in Arizona or New Mexico (J. Jaenike, pers. comm). In contrast, *D. occidentalis’* range is very restricted, as it is found only in certain regions of Southern and Baja California [24]. There are no recorded instances of sympatric populations of these species (J. Jaenike, pers. comm., K. Dyer, unpublished). Both of these species consume mushrooms as both larvae and adults, and they also use mushrooms as a mating substrate. The primary phenotypic difference between them is in abdominal pigmentation, as both male and female *D. suboccidentalis* harbor lighter abdominal pigmentation than *D. occidentalis* [25] (Additional file 1: Figure S1). Furthermore, these species also differ slightly in their metaphase chromosomes [26]. Very little genetic work has been completed on these species, but phylogenetic analyses show that they are more closely related to each other than to any other known *Drosophila* species [22,27] (Dyer, unpublished). Based on 14 autosomal protein-coding loci, synonymous divergence between *D. suboccidentalis* and *D. occidentalis* is about 0.01 substitutions/site (Dyer, unpublished data), which is similar to the genetic divergence seen among the species in the *D. simulans - D. sechellia - D. mauritiana* clade [28]. We estimate that *D. suboccidentalis* and *D. occidentalis* shared a common ancestor roughly 350,000 years ago, assuming 10 generations/year and a mutation rate of 2.8 × 10^−9^ subs/site/year per [29].

In this study, we examine individual components of reproductive isolation between *D. suboccidentalis* and *D. occidentalis*, including both premating and postmating barriers. Previous work suggested that hybrids produced by either reciprocal cross are viable and fertile [30]. We first use no-choice mating trials to quantify the prezygotic barriers to hybridization. We then use F1 hybrids to ask whether female discrimination is inherited in a recessive or dominant manner, and whether F1 hybrid males are able to attract pure species mates at a rate similar to pure species individuals. Second, we determine if any postmating, prezygotic barriers exist between these species by measuring egg production of females that mated with heterospecific males compared to those mated with conspecific males. Finally, we test for postzygotic isolation by quantifying hybrid female fecundity and hybrid male fertility.

## Results

### Premating barriers

**Figure 1:**
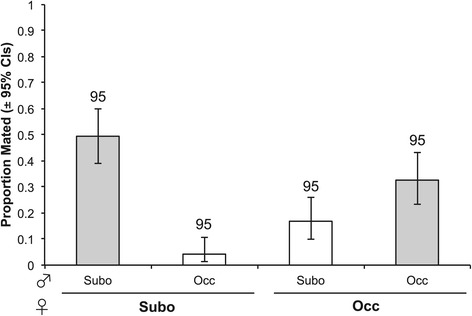
**Pure species mating rates.** Mating rates within and between species in no-choice trials. Error bars indicate the binomial 95% confidence intervals. “Subo” refers to *D. suboccidentalis* and “Occ” refers to *D. occidentalis*. Ninety-fine trials were completed for each type.

First, we find that the strength of premating isolating barriers is asymmetric between species (Figure 1). We performed no-choice mate trials in which a single female from either species was presented with a heterospecific or conspecific male. We found that male species (Likelihood ratio test [LRT]: ${\upchi}_1^2=14$, *P* = 0.0002) and the female x male species interaction (${\upchi}_1^2=52$, *P* < 0.0001) had significant effects on mating rate, whereas female species (${\upchi}_1^2=1.7$, *P* = 0.19) and block (${\upchi}_1^2=2.2$, *P* = 0.14) did not. This interaction is likely due to the females of both species mating at a lower rate with heterospecific males relative to conspecifics, indicating the presence of premating isolation. This pattern is especially striking for *D. suboccidentalis* females, which mate with *D. occidentalis* males 10-fold less often than with conspecific males (${\upchi}_1^2=47$, *P* < 0.0001). In contrast, *D. occidentalis* females mate with heterospecific males about half as often as with conspecifics, which is still a significant decrease (${\upchi}_1^2=5.5$, *P* = 0.019). Among heterospecific crosses only, *D. occidentalis* females mated more often with *D. suboccidentalis* males than *D. suboccidentalis* females mated with *D. occidentalis* males (${\upchi}_1^2=5.5$, *P* = 0.005). Among all successful matings, there was no difference in the copulation latency if the pair was of the same or different species (Wilcoxon rank sum test ${\upchi}_1^2=0.2$, *P* = 0.88; Additional file 1: Figure S2).

**Figure 2:**
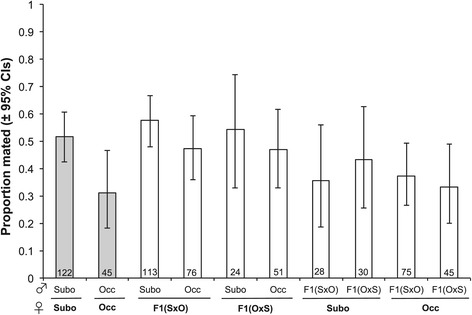
**Mating rates of crosses involving hybrids.** Mating rates of hybrids relative to pure species crosses in no-choice mate trials. Error bars are the 95% binomial confidence intervals, and the sample size is indicated within each bar. “Subo” refers to *D. suboccidentalis*, “Occ” refers to *D. occidentalis*, “F1(OxS)” are F1 hybrids from a *D. occidentalis* female, and “F1(SxO)” are F1 hybrids from a *D. suboccidentalis* female.

Next, we find that F1 hybrid females will mate with males of either species (Figure 2). Hybrids from either reciprocal cross mate with *D. suboccidentalis* males at a similar rate (${\upchi}_1^2=0.9$, *P* = 0.76), and this is similar to the mating rate observed between pure species *D. suboccidentalis* females and males (${\upchi}_1^2=0.3$, *P* = 0.56). The hybrid females from the two reciprocal crosses also mate with *D. occidentalis* males at a similar rate (${\upchi}_1^2=0.001$, *P* = 0.97), and these hybrids mate at a slightly higher rate with these males than do conspecific females (${\upchi}_1^2=3.6$, *P* = 0.059; Figure 2). These results suggest that the female premating discrimination has a recessive genetic basis, and is not due to a factor in the cytoplasm. Combining females from the two reciprocal crosses, we find that hybrid females mate at a similar rate with *D. suboccidentalis* and *D. occidentalis* males (${\upchi}_1^2=2.5$, *P* = 0.11). Finally, among the pairs that mated, the latency to copulation is similar in conspecific crosses and crosses involving hybrid females (Wilcoxon rank sum test ${\upchi}_1^2=.007$, *P* = 0.93; Additional file 1: Figure S3).

Finally, we find that pure species females accept F1 hybrid males at a rate similar to conspecific controls. Females of both species will mate with hybrid males from both reciprocal crosses (*D. occidentalis*: ${\upchi}_1^2=0.2$, *P* = 0.66, *D. suboccidentalis*: ${\upchi}_1^2=0.4$, *P* = 0.55), suggesting the male signal trait is probably not located on the X-chromosome. Furthermore, both *D. occidentalis* and *D. suboccidentalis* females will mate with hybrid males at a rate that is similar to pure species males (*D. occidentalis*: ${\upchi}_1^2=0.3$, *P* = 0.61, *D. suboccidentalis*: ${\upchi}_1^2=2.2$, *P* = 0.14), which suggests the male signal trait(s) has a dominant genetic basis. Among pairs that successfully mated, there was no significant difference in the latency to copulation of pure species pairs compared to pairs with hybrid males (Wilcoxon rank sum test ${\upchi}_1^2=.007$, *P* = 0.60).

### Postmating barriers

**Figure 3:**
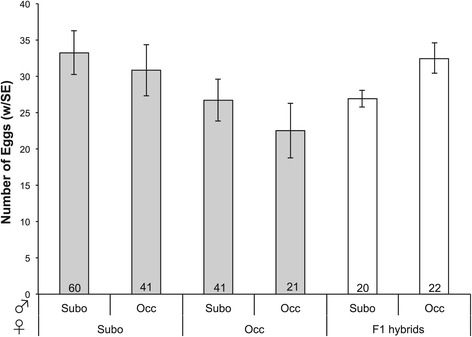
**Female egg production.** Female egg production after mating with conspecific or heterospecific males. Error bars represent standard errors, and the sample size of each type is shown within bar. “Subo” refers to *D. suboccidentalis*, “Occ” refers to *D. occidentalis*, and F1 hybrids combine the results from both reciprocal crosses.

**Figure 4:**
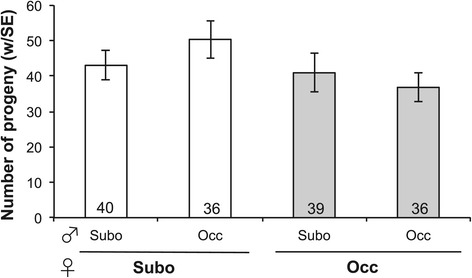
**Female progeny production.** Number of progeny produced by pure species females after conspecific or heterospecific matings. Bars indicate standard errors, and numbers within the bars are sample sizes. “Subo” refers to a *D. suboccidentalis* and “Occ” refers to *D. occidentalis*.

We do not find evidence for decreased reproductive output upon interspecific mating. Females mated with a conspecific male do not produce significantly more eggs than females mated with a heterospecific male (Wilcoxon rank sum test ${\upchi}_1^2=4.0$, *P* = 0.27; Figure 3 grey bars; all post-hoc pairwise tests *P* > 0.5). When we counted the offspring produced by pure species females that mated with conspecific or heterospecific males, we did not find a significant difference between conspecific and heterospecific crosses (Wilcoxon rank sum test ${\upchi}_1^2=3.6$, *P* = 0.31; Figure 4). For both *D. suboccidentalis* and *D. occidentalis* females, heterospecific crosses produced slightly more offspring than conspecific crosses, though none of the pairs of mating types were significantly different (all *P* > 0.3).

We find that hybrid females have a similar or higher fecundity as pure species females. Combining hybrid females from the two reciprocal crosses, these females produced about the same number of eggs as pure *D. suboccidentalis* females when mated to *D. suboccidentalis* males (Wilcoxon rank sum test ${\upchi}_1^2=0.5$, *P* = 0.48), but more eggs than pure species *D. occidentalis* females when mated to *D. occidentalis* males (Wilcoxon rank sum test ${\upchi}_1^2=6.6$, *P* = 0.01; Figure 3).

**Figure 5:**
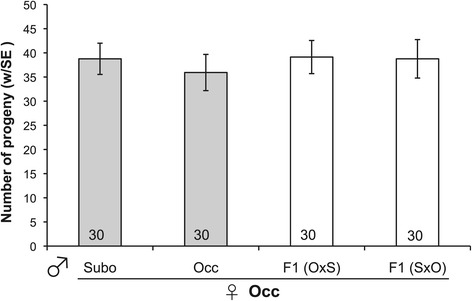
**Male progeny production.** Offspring production of hybrid males relative to pure species and heterospecific crosses. Error bars indicate standard errors, and the numbers within the bars are the sample sizes. “Subo” refers to *D. suboccidentalis*, “Occ” refers to *D. occidentalis*, “F1(SxO)” are F1 hybrids from a *D. suboccidentalis* female, and “F1(OxS)” are F1 hybrids from a *D. occidentalis* female.

Even though effects on male fertility are often the first type of postzygotic isolation to arise [31], we identified no postzygotic effects on male fertility in heterospecific crosses between *D. suboccidentalis* and *D. occidentalis*. First, using assays of sperm motility, we found that every assayed pure species as well as F1 hybrid male from both reciprocal crosses produced fully motile sperm (*N* = 20 for each type). Second, F1 hybrid males from either reciprocal cross sired a similar number of offspring as either type of pure species male (*F*_3,116_ = 0.17, *P* = 0.92; Figure 5; all post-hoc pairwise tests *P* > 0.5).

## Discussion

*D. suboccidentalis* and *D. occidentalis* were determined to be different species based on their geographic locations, chromosomal patterns, and slight morphological differences [25]. Here, we find substantial premating isolation but no postmating isolation between these closely related species. First, females of both *D. suboccidentalis* and *D. occidentalis* discriminate against mating with males of the opposite species, though this pattern is much stronger for *D. suboccidentalis* females. During our no-choice mate trials, males of both species vigorously court females of either species (N. Arthur, personal observation). When heterospecific matings do occur, we do not see a decrease in the production of eggs or viable offspring relative to conspecific males, indicating a lack of postmating prezygotic isolating barriers. Finally, the hybrid offspring appear to be vigorous and both sexes are fully fertile, suggesting a lack of intrinsic postzygotic isolating mechanisms between these species, and confirming qualitative results of a previous study [30]. We note that our experiments used flies from one population of each species, and thus our study does not capture the variation that may occur in reproductive isolation between *D. suboccidentalis* and *D. occidentalis*. Further studies are necessary to ask if the patterns we observe are found from flies collected from other geographic locations. Furthermore, our experiments used several isofemale lines of *D. occidentalis* but a single yet genetically diverse stock of *D. suboccidentalis*, and there may be additional genetic variation present within populations that we have not identified here.

It is perhaps surprising that allopatric populations of such closely related species have evolved prezygotic isolating barriers in the absence of intrinsic postzygotic isolation. To compare the level of premating isolation between *D. suboccidentalis* and *D. occidentalis* with other species pairs, we calculated the strength of sexual isolation using the same metric used by Coyne and Orr [20,21]. The index is calculated as 1-(frequency of heterospecific matings)/(frequency of conspecific matings), where a value of 1 indicates pure assortative mating and a value of 0 indicates random mating between species. Using data from our no-choice experiments, we obtained a sexual isolation index value of 0.744 between *D. suboccidentalis* and *D. occidentalis*. The synonymous divergence between these species is ~ 0.01 substitutions/site (Dyer, unpublished), which is similar to other *Drosophila* species pairs with a Nei’s *D* of ~ 0.3 (e.g. [28]). Thus we can ask how sexual isolation in this species pair compares to others with similar divergence. Considering only species pairs with Nei’s *D* of 0.25-0.35 in the Coyne and Orr data as updated by Yukilevich [32], the sexual isolation between *D. suboccidentalis* and *D. occidentalis* is stronger than most other allopatric pairs (mean isolation index of 0.67, with 3 of the 11 pairs showing stronger isolation). In contrast, the sexual isolation between *D. suboccidentalis* and *D. occidentalis* is relatively low compared to sympatric pairs (mean isolation index of 0.85, with 10 of the 12 pairs showing stronger isolation). Thus, *D. suboccidentalis* and *D. occidentalis* appear to be somewhat more sexually isolated than expected based on their current geographic distributions.

Asymmetry in sexual isolation between *Drosophila* species has been observed in many previous studies [1,32-38]. A common explanation for asymmetric premating isolation is that hybrid fitness is lower in one cross direction than the other, which is expected to result in a stronger selective pressure on the females from the species with lower hybrid fitness to avoid heterospecific matings. In our studies of *D. suboccidentalis* and *D. occidentalis* we do not identify any intrinsic postmating isolation in either cross direction, but we have not tested the ecological fitness of the hybrids, which if low could select for premating discrimination. The patterns of mate discrimination we observe may also be caused by differences in premating conditions. For instance, females from the species with the smaller population size may encounter heterospecific males at a high rate and thus have a relatively greater cost of hybridization [32,39]. This would predict that *D. occidentalis*, the species with the more restricted range, should be more choosy. However, we find that *D. suboccidentalis*, which has a broader range, to be the more choosy of these species.

We speculate that *D. suboccidentalis* females may have evolved stronger mate discrimination due to interactions with species other than *D. occidentalis* [40]. While *D. occidentalis* has a limited range and may interact with few other species, *D. suboccidentalis* co-occurs with many other quinaria group species. For instance, in the northern part of the range this includes *D. recens*, *D. subquinaria*, *D. rellima*, and *D. falleni*. Character displacement with other closely related sympatric species in such traits as courtship songs and cuticular hydrocarbon profiles could explain how premating isolation with *D. occidentalis* can be strong even though postzygotic isolation is absent. In this scenario, discrimination against *D. occidentalis* would result as a byproduct of selection rather than a direct result of it. We note that in the laboratory *D. suboccidentalis* can hybridize with other quinaria group species in addition to *D. occidentalis*, and in our assays the hybrid males of these other crosses are sterile [24,27] (K. Dyer, unpublished). While *D. suboccidentalis* has not been found recently in the southwestern part of North America, if it does occur there it would be interesting to not only test females from this part of the range for discrimination against *D. occidentalis*, but also against males from northern conspecific populations, and vice versa.

The sexual isolation that occurs between pure species individuals is lost when hybrids are used. Our results suggest female premating discrimination has a recessive genetic basis, which has also been observed in other *Drosophila* species (e.g. [41-43]). One may expect that recessive female preference alleles could be more likely to spread in allopatry than in sympatry, as in sympatry the hybrid offspring could mate with males from either pure species and ultimately prevent the evolution of assortative mating [1]. Furthermore, if female mate preferences evolve as a pleiotropic byproduct of an ecological adaptation rather than as a result of direct selection, when these preference alleles are recessive they may be driven to a higher frequency faster than when they are dominant [44]. Ultimately, careful genetic dissection is necessary to understand the selective forces, if any, that drove the changes in female preferences we observe here.

Further work is also necessary to determine the male traits that are used as mating signals in this system, and whether the same signals are used by females of each species. The acceptance of hybrid males from either reciprocal cross by females of either species suggests that the male signal trait(s) has a dominant autosomal genetic basis. Hybrid males have intermediate pigmentation phenotypes relative to pure species males (N. Arthur, personal observation), thus species-specific pigmentation cues may not be essential for females to mate. Other male signal traits that often differ between closely related species include epicuticular pheromones and songs (reviewed in [45,46]).

In summary, we expect that if matings do occur between these species, there would be few intrinsic barriers to gene flow between them. The hybrids do not appear to suffer any intrinsic postzygotic effects, and they also benefit from being accepted by either species. Thus, introgression of genetic material could occur through backcrossing in either direction. A thorough examination of genetic differentiation between these species has not been completed, but we expect that if the effective population sizes are high, as we have found in other quinaria group species (e.g. [47,48]), the lineages have probably not become reciprocally monophyletic in the short time since they diverged.

## Conclusions

It is important to quantify both pre and postmating reproductive barriers to determine the mechanisms that promote and maintain reproductive isolation between species. We find that the recently diverged species *D. suboccidentalis* and *D. occidentalis* show substantial sexual isolation. Furthermore, this strength of this isolation is asymmetric, as *D. suboccidentalis* females discriminate strongly against *D. occidentalis* males whereas *D. occidentalis* discriminate only moderately against *D. suboccidentalis* males. These premating barriers have formed in spite of a lack of intrinsic postzygotic barriers and even though these species are thought to be allopatric in geographic range. We propose that differences in the encounter rate with other closely related species might underlie this pattern. Alternatively, ecological fitness of the hybrids may also be lower in one direction, which could also select for increased premating barriers. These results lay the groundwork for future studies to dissect the geographic variation in reproductive isolation as well as the phenotypic and genetic mechanisms that underlie the reproductive barriers between these species. These can be combined with population genetic studies that inform the extent of gene flow and the consequences of geographic isolation. Ultimately, these mechanistic studies will inform the roles of natural and sexual selection and how they interact to drive divergence between these lineages.

## Methods

### *Drosophila* strains and rearing

Flies used in this study were derived from a population of each species that were allopatric with one another. For *D. suboccidentalis* we used a stock in which 12 isofemale lines collected in 2010 near Shuswap, British Columbia, were mixed together about two years before the experiments commenced. For *D. occidentalis* we used flies from 10 isofemale lines collected near Idyllwild, CA, in 2010. In the assays described below, in conspecific trials *D. occidentalis* were mated with flies from their own line; in these and all other assays we tested for line effects but did not find any for any of our experiments (all *P* > 0.05), thus the data were combined across lines in the analyses. We note that there may be a difference in inbreeding between the stocks of the two species, which may result in a lower overall productivity of *D. occidentalis*. Wild-caught flies were identified to species using morphological characteristics [25], and were also verified using molecular markers. Specifically, we sequenced a son from each of six wild-caught females from each species at the Y-linked gene *kl-3*. We found that within each species sequences were identical across 757 bp of coding sequence, but that there were two synonymous fixed differences between species. Furthermore, comparing these *kl-3* sequences to other quinaria group species [49] (K. Dyer, unpublished) revealed that these species are more closely related to each other than they are to any other species for which we have data.

Flies were maintained on Instant *Drosophila* Medium (Carolina Biological, Burlington, NC, USA) supplemented with commercial mushroom *Agaricus bisporus*, and reared at 20°C with 12 hour light/dark cycles and 60% relative humidity. Virgins were collected using light CO_2_ anesthesia, held at a density of fewer than 20 flies per vial, and were at least 4 days old and up to 7 days old when used in experiments. We used sexually mature but young flies to be sure that any discrimination behaviors would be evident.

### Premating barriers

We used no-choice mate trials to quantify patterns of premating isolation. In all of these experiments, 4-day old virgin females were transferred individually using light CO_2_ anesthesia into small vials (4.5 cm long × 1 cm diameter) that contained a blended mushroom-agar medium, and given at least 12 hours to recover from any effects of the anesthesia. The next day, within one hour of the incubator lights on, a single 4-day old male was added to each vial. We observed each vial for 2 hours and noted whether copulation occurred; if it did we noted the latency to copulation, or the time from when the male was introduced to when copulation commenced.

We first paired females of each species with males of their own or the opposite species. We used an ordinal logistic regression to assess the effect of female species, male species, female x male interaction, and block on mating rates. These and all subsequent statistical analyses were completed using JMP version 10 (SAS Institute, Cary, NC). We then asked if females of each species mated with the opposite species less than with their own species, using a Pearson’s Chi-squared test. We also asked if *D. suboccidentalis* females mate with heterospecific males at a rate lower than *D. occidentalis* females mate with heterospecific males, again using a Pearson’s Chi-squared test. We used a Wilcoxon rank sum test to determine if there were differences in the latency to copulation between conspecific and heterospecific crosses.

We generated F1 hybrids in both reciprocal directions; females from the two reciprocal crosses differ only in their cytoplasm but are identical at their autosomes and X-chromosomes, whereas males differ in their X-chromosome and cytoplasm but their autosomes are identical. We conducted no-choice mating trials where a virgin hybrid F1 male or female was paired with a virgin pure species individual of the opposite sex. Both types of pure species matings were completed as controls. We first asked if hybrid females accept mating with males of each species. For each male species we compared the mating rates of the hybrid females to those of conspecific females using an ordinal logistic regression with pure or hybrid female and cross direction nested within pure or hybrid female as the effects in the model. We also used an ordinal logistic regression to ask whether hybrid females from the two reciprocal crosses mated at similar rates with males of each species.

We analyzed the hybrid male mating rates separately by the female species they were paired with. Within each female species (*D. suboccidentalis* or *D. occidentalis*) we used an ordinal logistic regression with pure or hybrid male and cross direction nested within pure or hybrid male as the effects in the model. This will address whether hybrid males obtain matings at different rates than pure species males, as well as if the males from the two reciprocal crosses obtain different rates of matings. We also used Wilcoxon rank sum tests to analyze the latency to copulation, which we conducted separately for hybrid males compared to pure species controls, and for hybrid females compared to pure species controls.

### Postmating barriers: female egg and progeny production

We asked if pure species females produce fewer eggs after mating with a heterospecific male than a conspecific male, and if hybrid females produce fewer eggs than pure species females. We placed 4-day old virgin pure species or F1 hybrid females individually in vials containing blended mushroom-agar medium, and then added either a *D. suboccidentalis* or *D. occidentalis* male. Hybrid females from both reciprocal crosses were used. Females that were observed to mate were placed in fresh vials and allowed to lay eggs for three days, after which the female was removed and the number of eggs in each vial was counted. To compare the number of eggs produced after conspecific vs. heterospecific matings, we used a Wilcoxon rank sum test and the Steel-Dwass nonparametric method for post-hoc pairwise comparisons. To compare the number of eggs produced by F1 hybrid females, we first asked whether the females from each reciprocal cross were similar in egg production when crossed to each male species, using a Wilcoxon rank sum test. Because they were similar (each *P* > 0.1), we combined the data from each reciprocal cross. We then used a Wilcoxon rank sum test to ask whether hybrids lay fewer eggs than conspecific females after mating with each male species.

We also asked whether pure species females produce fewer viable offspring when crossed with heterospecific versus conspecific males. The methods were similar to the egg counts, except in this case mated females remained individually in regular food vials for one week, after which they were removed and all of the offspring were counted. We tested for variation in the number of offspring among the cross types with a Wilcoxon rank sum test and the Steel-Dwass nonparametric method for post-hoc comparisons.

### Postmating barriers: male fertility

We tested whether sperm were motile in F1 hybrid males relative to pure species controls. F1 hybrid males from both reciprocal crosses as well as pure species males were collected as virgins and allowed to age for four days. The testes were removed in Ringer’s solution, and an incision was made in the seminal vesicle to allow the mature sperm to be removed. Using a compound microscope, we categorized the mature sperm as fully motile, partially motile, or non-motile (for a full description, see [50]).

To test the fertility of F1 hybrid males relative to pure species controls, we placed 4-day old virgin F1 hybrid or pure species males individually in vials that contained three 4-day-old *D. occidentalis* females. Hybrid F1 males from both reciprocal crosses were used. *D. occidentalis* females were used as tester females because these females are much less discriminatory than *D. suboccidentalis* females. The flies were left together for 2 days, after which the male was discarded, and the females were allowed to lay eggs for an additional 4 days. We counted all of the progeny that emerged from each vial. We used a one-way analysis of variance to assess the effect of male type on offspring production, and then we used post-hoc *t*-tests to test each pairwise comparison.

### Availability of supporting data

The data sets supporting the results of this article are available in the Dryad.org repository, http://dx.doi.org/10.5061/dryad.tj57h.

## Additional file

Additional file 1:
**Figure S1.** Phenotypic Differences Between the Species. Representative female and male individuals from *Drosophila suboccidentalis* (top) and *Drosophila occidentalis* (bottom), highlighting the difference in abdominal pigmentation between the two species. **Figure S2.** Latency to copulation for each type of pure species cross. Only pairs that copulated are included. Horizontal bars indicate the median for each cross, and boxes represent upper and lower quartiles. “Subo” refers to *D. suboccidentalis* and “Occ” refers to *D. occidentalis*. **Figure S3.** Latency to copulation for crosses involving hybrid individuals, compared to pure species controls. Only pairs that copulated are included. Horizontal bars indicate the median for each cross, and boxes represent upper and lower quartiles. “Subo” refers to *D. suboccidentalis*, “Occ” refers to *D. occidentalis*, “F1(OxS)” refers to a F1 hybrid from a *D. occidentalis* female, and “F1(SxO)” refers to a F1 hybrid from a *D. suboccidentalis* female.
