# Supplementary material for: Asymmetrical sexual isolation but no postmating isolation between the closely related species Drosophila suboccidentalis and Drosophila occidentalis
Source: BMC Evol Biol. 2015 Mar 12;15:38. doi: 10.1186/s12862-015-0328-y (PMC4369358; doi:10.1186/s12862-015-0328-y)

Supplemental Figure 1. Phenotypic Differences Between the Species. Representative female and male individuals from *Drosophila suboccidentalis* (top) and *Drosophila occidentalis* (bottom), highlighting the difference in abdominal pigmentation between the two species.

D. suboccidentalis female *D. suboccidentalis* male

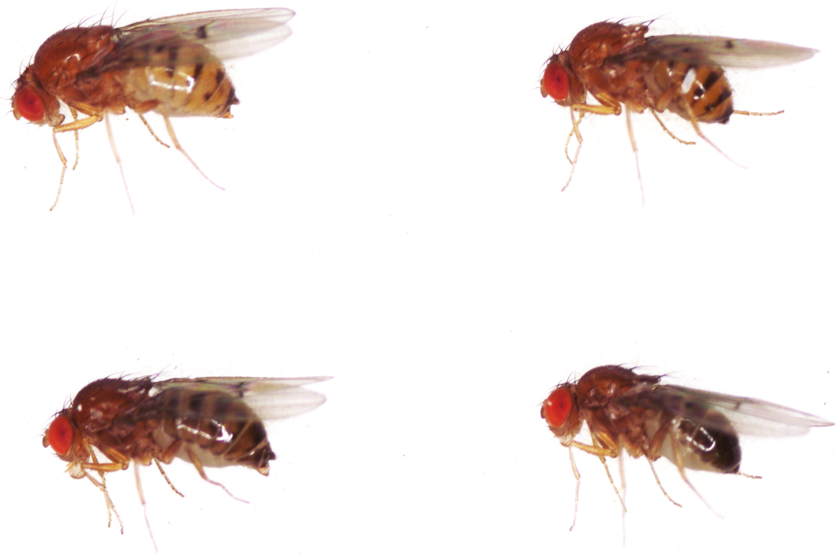

D. occidentalis female *D. occidentalis* male

Supplemental Figure 2. Latency to copulation for each type of pure species cross. Only pairs that copulated are included. Horizontal bars indicate the median for each cross, and boxes represent upper and lower quartiles. "Subo" refers to *D. suboccidentalis* and "Occ" refers to *D. occidentalis.*

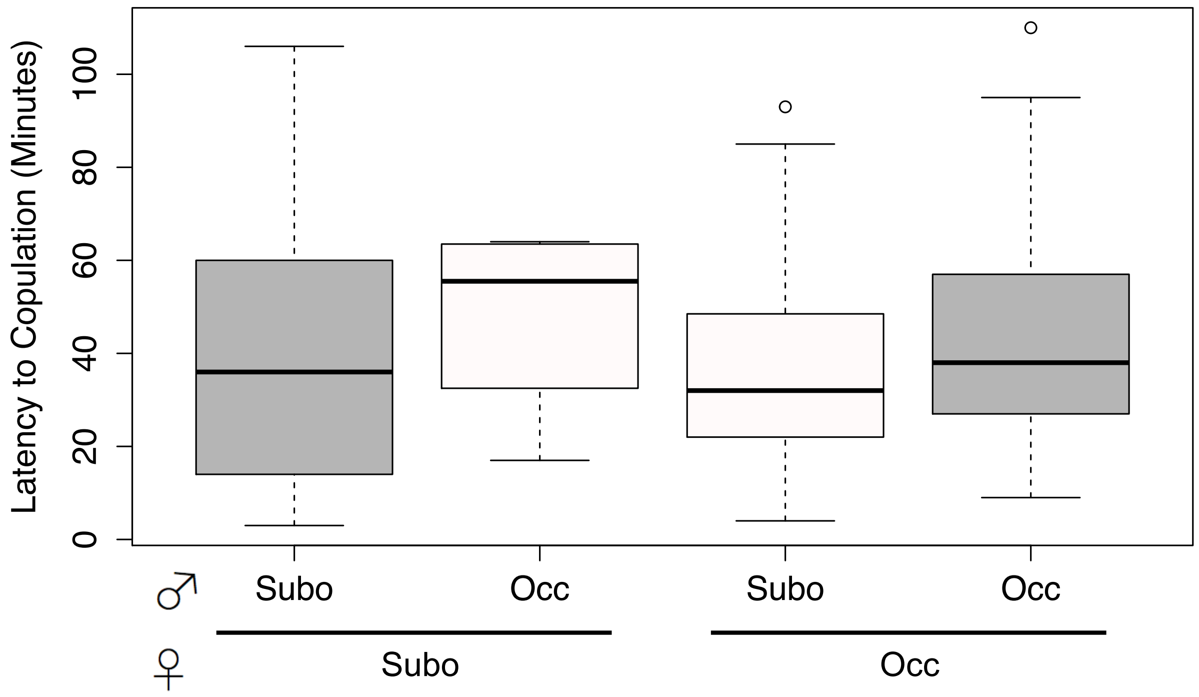

Supplemental Figure 3. Latency to copulation for crosses involving hybrid individuals, compared to pure species controls. Only pairs that copulated are included. Horizontal bars indicate the median for each cross, and boxes represent upper and lower quartiles. "Subo" refers to *D. suboccidentalis,* "Occ" refers to *D. occidentalis,* "F1(OxS)" refers to a F1 hybrid from a *D. occidentalis* female, and "F1(SxO)" refers to a F1 hybrid from a *D. suboccidentalis* female.

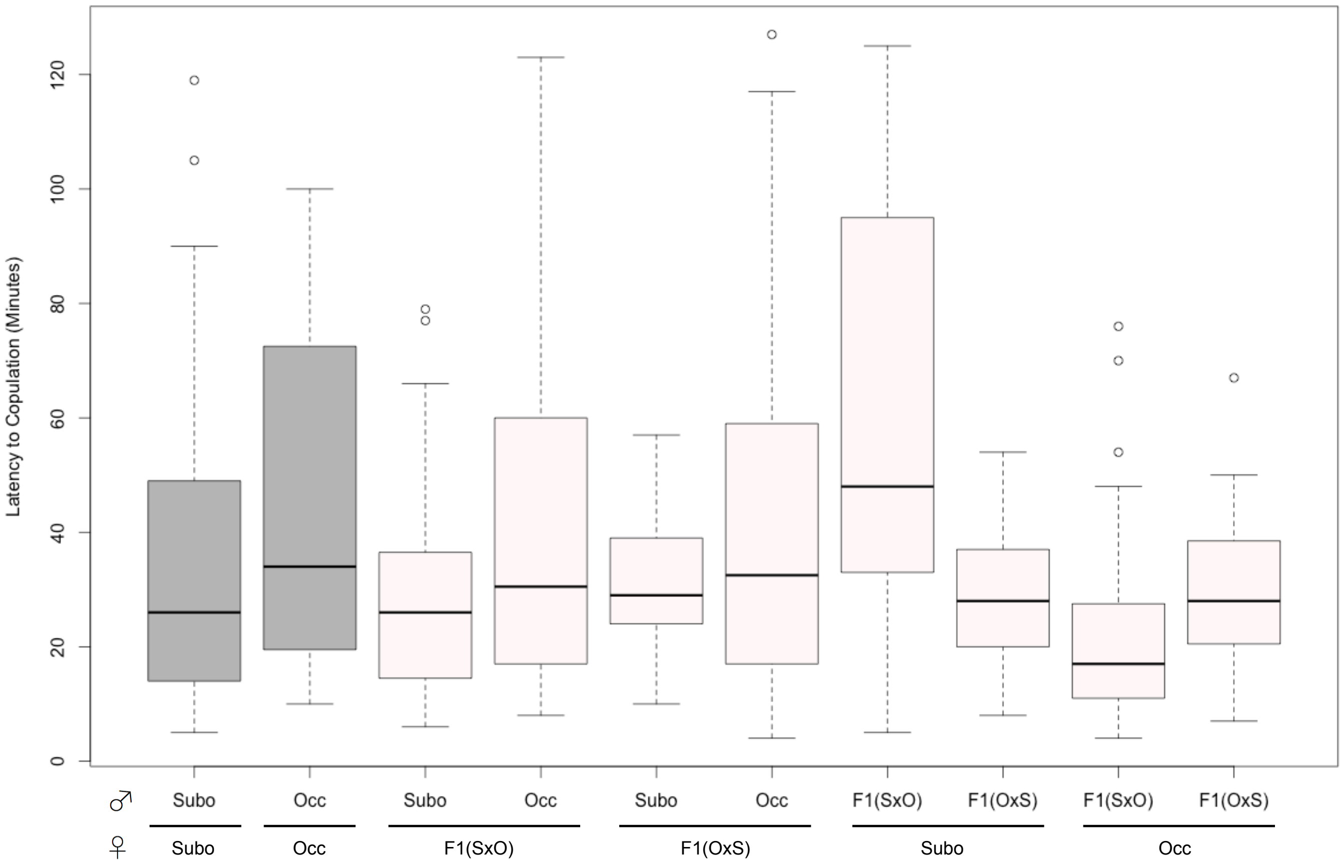

Supplement: Additional file 1: — Figure S1. Phenotypic Differences Between the Species. Representative female and male individuals from Drosophila suboccidentalis (top) and Drosophila occidentalis (bottom), highlighting the difference in abdominal pigmentation between the two species. Figure S2. Latency to copulation for each type of pure species cross. Only pairs that copulated are included. Horizontal bars indicate the median for each cross, and boxes represent upper and lower quartiles. “Subo” refers to D. suboccidentalis and “Occ” refers to D. occidentalis. Figure S3. Latency to copulation for crosses involving hybrid individuals, compared to pure species controls. Only pairs that copulated are included. Horizontal bars indicate the median for each cross, and boxes represent upper and lower quartiles. “Subo” refers to D. suboccidentalis, “Occ” refers to D. occidentalis, “F1(OxS)” refers to a F1 hybrid from a D. occidentalis female, and “F1(SxO)” refers to a F1 hybrid from a D. suboccidentalis female. [file 12862_2015_328_MOESM1_ESM.pdf]
